# A multicentre cross-sectional survey study on acute wound classification in the emergency department and its interobserver variability

**DOI:** 10.1038/s41598-022-13221-1

**Published:** 2022-06-14

**Authors:** Lisanne van Gennip, Frederike J. C. Haverkamp, Özcan Sir, Edward C. T. H. Tan

**Affiliations:** 1 Department of Surgery, Radboudumc, P.O. Box 9101, 6500 HB Nijmegen, The Netherlands; 2 Department of Emergency Medicine, Radboudumc, 6500 HB Nijmegen, The Netherlands

**Keywords:** Health care, Medical research

## Abstract

Annually, a vast number of patients visits the emergency department for acute wounds. Many wound classification systems exist, but often these were not originally designed for acute wounds. This study aimed to assess the most frequently used classifications for acute wounds in the Netherlands and the interobserver variability of the Gustilo Anderson wound classification (GAWC) and Red Cross wound classification (RCWC) in acute wounds. This multicentre cross-sectional survey study employed an online oral questionnaire. We contacted emergency physicians from eleven hospitals in the south-eastern part of the Netherlands and identified the currently applied classifications. Participants classified ten fictitious wounds by applying the GAWC and RCWC. Afterwards, they rated the user-friendliness of these classifications. We examined the interobserver variability of both classifications using a Fleiss’ kappa analysis, with a subdivision in RCWC grades and types representing wound severity and injured tissue structures. The study included twenty emergency physicians from eight hospitals. Fifty percent of the participants reported using a classification for acute wounds, mostly the GAWC. The interobserver variability of the GAWC (κ = 0.46; 95% CI 0.44–0.49) and RCWC grades (κ = 0.56; 95% CI 0.53–0.59) was moderate, and it was good for the RCWC types (κ = 0.69; 95% CI 0.66–0.73). Participants considered both classifications helpful for acute wound assessment when the emergency physician was less experienced, despite a moderate user-friendliness. The GAWC was only of additional value in wounds with fractures, whereas the RCWC’s additional value in acute wound assessment was independent of the presence of a fracture. Emergency physicians are reserved to use a classification for acute wound assessment. The interobserver variability of the GAWC and RCWC in acute wounds is promising, and both classifications are easy to apply. However, their user-friendliness is moderate. It is recommended to apply the GAWC to acute wounds with underlying fractures and the RCWC to major traumatic injuries. Awareness should be raised of existing wound classifications, specifically among less experienced healthcare professionals.

## Introduction

Approximately 176,000 patients visited the Dutch emergency departments (EDs) with acute open wounds in 2016^1^. Wound assessment should be adequately performed to determine wound severity and guide wound management^2^. As there is no standard classification in practice for acute wound assessment, wound description is currently not performed uniformly. This was also observed with skin tears, for which a need was observed for simplified documentation and assessment methods among healthcare providers^3^.

The Dutch guideline on wound care advocates classifying wounds according to the degree of contamination^4^. This requires information on the type of injury and time since the injury occurred. The Red Yellow Black (RYB) system, applied by nurses to the care of (chronic) wounds^5^, can be combined with wound moistness to evaluate acute wounds^4^. Additionally, the TIME model is advocated to aid a uniform and systematic approach to wound care^4,6^, as its four parameters, including the type of Tissue affected, presence of Infection, Moistness of the wound, and aspect of wound Edges are systematically assessed. Classifications originally designed for assessing acute soft tissue injuries with underlying fractures are the Gustilo Anderson wound classification (GAWC) (Additional file 1)^7^, AO soft tissue classification^8^, OTA Open Fracture classification^9^, and Tscherne classification^10^. Their corresponding interobserver variability was studied in wounds with fractures to ensure uniform application of these classifications and was shown to vary from moderate to good^11–14^. Finally, the Red Cross wound classification (RCWC) (Additional file 2) can be applied to acute wounds with or without an underlying fracture. Although it was originally designed for war wounds^15^, it may be a valuable alternative for assessing acute wounds within civilian healthcare.

Although many classification systems exist to assess wounds, these are generally not designed for acute wounds and do not appear to have been incorporated in daily practice in the ED. Using a classification could aid less experienced medical professionals during their physical examination and patient care, as it offers a systematic approach to wound assessment and draws attention to the wound. Additionally, implementing a standard and reliable classification system for acute wound assessment in the ED will provide uniform wound description. This promotes clear communication and facilitates scientific research. Therefore, this study aimed to identify which classification is currently used most frequently for assessing acute wounds in Dutch EDs, and evaluate the interobserver variability of the GAWC and RCWC in acute wounds.

## Methods

This multicentre cross-sectional survey was performed as an online oral questionnaire (Additional file 3). The aim of this study was to identify which classification is currently used most frequently for assessing acute wounds in Dutch EDs, and to evaluate the interobserver variability of the GAWC and RCWC in acute wounds. The tripartite questionnaire was conducted using video or phone calls due to the COVID-19 pandemic restrictions. This study was deemed exempt from the Medical Research Involving Human Subjects Act by the Radboudumc Medical Ethics Committee and has been performed in accordance with the relevant guidelines. Subjects voluntarily signed up to participate by replying to our invitation e-mail. Study participants were informed about the research and provided informed consent before commencing with the questionnaire.

The study population comprised emergency physicians and emergency medicine residents from eleven hospitals in the south-eastern part of the Netherlands. The following hospitals were contacted: Bernhoven (Uden), Maashospital Pantein (Boxmeer), Hospital Rivierenland (Tiel), Jeroen Bosch hospital (Den Bosch), Elisabeth TweeSteden hospital (Tilburg), Rijnstate (Arnhem), Slingeland hospital (Doetinchem), Regional hospital Koningin Beatrix (Winterswijk), Hospital Gelderse Vallei (Ede), Canisius Wilhelmina hospital (Nijmegen), and Radboudumc (Nijmegen). One contact person per hospital was asked to forward our request for study participation. All included participants provided informed consent. Participation was voluntary, and withdrawal was possible at any time until completion of the questionnaire, as participant-specific data was then no longer traceable. Therefore, the database had no missing values. Before starting the oral questionnaire, participants received background information and an example of a wound description performed with the GAWC and RCWC.

The first part of the questionnaire comprised the collection of participants’ characteristics, multiple-choice questions about the currently most used classification for acute wounds in the ED, and an open question asking why this classification is used. For the second part, ten fictitious patient cases of acute wounds were compiled from open-source pictures and radiographs derived from study books and the internet, with an equal number of cases with and without fractures. All cases comprised a short patient history, picture of the wound, radiograph (if applicable), and other information required to classify the wounds using the GAWC and RCWC. A trauma surgeon of the Radboudumc reviewed all fictitious patient cases on accuracy and relevance. Although burns are considered acute wounds, they were excluded, as they are classified differently^16,17^, and are often excluded from acute wound management guidelines^4^. Participants evaluated all ten patient cases, applying both classifications. The final part of the questionnaire comprised the participants’ opinions about the user-friendliness of these classifications, rating them on a scale from one to five (one being ‘strongly disagree’ and five being ‘strongly agree’). The wound evaluations were performed independently from other study participants, as the online oral questionnaires were held separately.

Collected data was anonymously recorded in Microsoft Office Excel® and transferred to an SPSS database in the Radboudumc on encrypted servers only accessible by individuals directly involved in this research. Participants’ characteristics recorded were as follows: hospital, position (emergency physician/emergency medicine resident), total years of experience as an emergency doctor, currently used classification and reason for use (if applicable), and familiarity with and frequency of use of the GAWC and RCWC. Variables recorded for the GAWC were as follows: energy transfer, wound size, soft tissue injury, contamination, fracture type, periosteal stripping, skin coverage method, neurovascular injury, and assigned wound type. Variables recorded for the RCWC included wound size, cavity, type of fracture, vital structure, metallic body, and assigned wound grade and type representing, respectively, wound severity and injured tissue structures^18–21^.

Descriptive and Fleiss’ kappa analyses were executed using SPSS statistical software (IBM SPSS Statistics for Windows, version 25.0 and IBM SPSS Statistics for MacOS, version 27.0, respectively). Descriptive analyses were performed for the participants’ background characteristics, reported most used classification, and participants’ opinions on user-friendliness. The results are presented as medians with interquartile ranges and percentages. A Fleiss’ kappa analysis^22^ was executed to calculate the interobserver variability for the GAWC types and RCWC types and grades in the total study population, as well as separately for emergency physicians and emergency medicine residents. The outcomes are presented as kappa values with 95% confidence intervals and p-values. A p-value of < 0.05 was considered significant. The guidelines of Landis and Koch^23^, based on the value of Cohen’s kappa coefficient, were used to assess the level of agreement with κ = 0.40–0.60 indicating moderate agreement; κ = 0.61–0.80 indicating good agreement; κ > 0.80 indicating very good agreement. Representativeness was considered good by an independent statistician as with the variety of the included hospitals major differences in other Dutch hospitals are not expected. Sample size calculation or power analysis was not indicated due to the non-comparative nature of this research. After statistical consultation, a sample size of 20 participants was proposed and was deemed to be sufficient.

### Ethics approval and consent to participate

This study was deemed exempt from the Medical Research Involving Human Subjects Act by the Radboudumc Medical Ethics Committee (No. 2020-6578). Participants provided informed consent by replying to our request for study participation.

## Results

In total, eight hospitals (72.7%; 8/11) in the south-eastern part of the Netherlands were included, resulting in the inclusion of twenty participants (Table 1). This led to the inclusion of twelve emergency physicians and eight emergency medicine residents employed in the participating hospitals, with a total response rate of 11.8% (12/102) and 5.9% (8/136), respectively. Non-participation was due to the COVID-19 pandemic and the accompanying high demand on patient care which was currently priority (Bernhoven, Uden) and other non-specified reasons for rejection on our request to participate (Maashospital Pantein, Boxmeer; Hospital Rivierenland, Tiel).

**Table 1:** Study population basic characteristics and experience with GAWC and RCWC.

	Emergency physicians	Emergency medicine residents	Total
Number of study participants (%)	12 (60.0%)	8 (40.0%)	20 (100%)
**Type of hospital, N (%)**^**a**^			
Level 1 hospital^b^	1 (8.3%)	1 (12.5%)	2 (10.0%)
Level 2 hospital^b^	10 (83.3%)	6 (75.0%)	16 (80.0%)
Level 3 hospital^b^	1 (8.3%)	1 (12.5%)	2 (10.0%)
Years of work experience, median (IQR)^c^	13.5 (11.3–18)	1.6 (0.5–3.9)	10.5 (2.8–16.5)
Use of wound classification before this study, N (%)^a^	8 (66.7%)	2 (25.0%)	10 (50.0%)
**Familiarity with, N (%)**^**a,d**^			
GAWC	8 (66.7%)	5 (62.5%)	13 (65.0%)
RCWC	2 (16.7%)	0 (0.0%)	2 (10.0%)
**Previously used, N (%)**^**a**^			
GAWC			
Never	4 (33.3%)	4 (50.0%)	8 (40.0%)
Less than once per week	7 (58.3%)	4 (50.0%)	11 (55.0%)
Once or more per week	1 (8.3%)	0 (0.0%)	1 (5.0%)
Daily	0 (0.0%)	0 (0.0%)	0 (0.0%)
RCWC			
Never	12 (100%)	8 (100%)	20 (100%)
Less than once per week	0 (0.0%)	0 (0.0%)	0 (0.0%)
Once or more per week	0 (0.0%)	0 (0.0%)	0 (0.0%)
Daily	0 (0.0%)	0 (0.0%)	0 (0.0%)

^a^Percentages are calculated based on the total number of participants of the column.

^b^Based on the trauma level criteria according to the American college of surgeons.

^c^Total years of experience as an emergency physician or emergency medicine resident.

^d^Familiarity with the classification before participation in this study.

Table 1 depicts the study population’s background characteristics and familiarity and experience with the GAWC and RCWC. Participants’ work experience varied widely, with an overall median of 10.5 years (IQR 2.8–16.5 years). One participant (5.0%; 1/20) reported using the GAWC more than once a week. None of the participants had used the RCWC before.

Fifty percent (10/20) of the participants use no classification when encountering acute wounds (Table 1). Some participants explained they do not need a classification system as they already assess and describe certain wound characteristics, which overlap with those incorporated in the GAWC. Wound characteristics mentioned included wound size, location and degree of contamination, presence or absence of a fracture, and neurovascular damage. Eight participants use the GAWC to support decision-making in treatment strategies (administration of antibiotics), derive prognostic information, or systematically assess wounds. Some participants additionally mentioned that the GAWC was included in the hospital protocol for open fracture assessment and is common practice. This was the case for two hospitals (2/8; 25.0%). The TIME model was the second most used classification and was used by one participant, as it is mentioned in the hospital’s quality portal. None of the participating hospitals have a protocol for acute wound assessment.

The results of the Fleiss’ kappa analyses of the GAWC and RCWC are listed in Table 2. The overall agreement was moderate for the GAWC types (κ = 0.46; 95% CI 0.44–0.49; p < 0.001) and RCWC grades (κ = 0.56; 95% CI 0.53–0.59; p < 0.001). Good overall agreement was found for the RCWC types (κ = 0.69; 95% CI 0.66–0.73; p < 0.001). Emergency medicine residents were in slightly better agreement (κ = 0.72) than emergency physicians (κ = 0.68) regarding the RCWC types. Among emergency physicians, agreement was almost good for the RCWC grades (κ = 0.59).

**Table 2:** Overall kappa (κ) values of the GAWC and RCWC.

	Types GAWC	Grades RCWC	Types RCWC
Emergency physicians (95% CI)^a^	0.45 (0.41–0.50)*	0.59 (0.54–0.65)*	0.68 (0.63–0.74)*
Emergency medicine residents (95% CI)^a^	0.47 (0.40–0.54)*	0.54 (0.46–0.62)*	0.72 (0.63–0.80)*
Total (95% CI)^a^	0.46 (0.44–0.49)*	0.56 (0.53–0.59)*	0.69 (0.66–0.73)*

^a^Lower and upper bound of 95% confidence interval.

*P-value < 0.05 indicating statistical significance.

**Figure 1:**
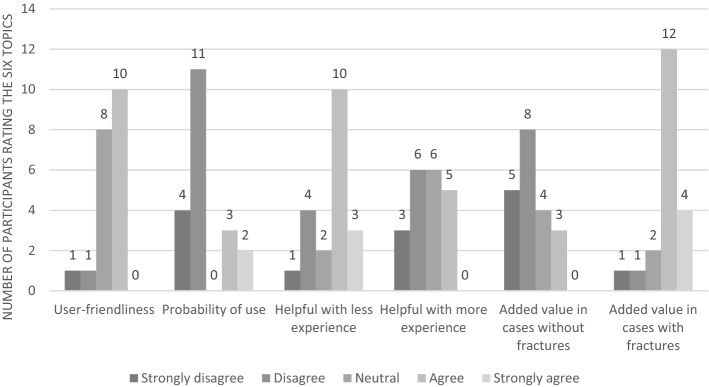
Overview of rated opinion of the GAWC.

**Figure 2:**
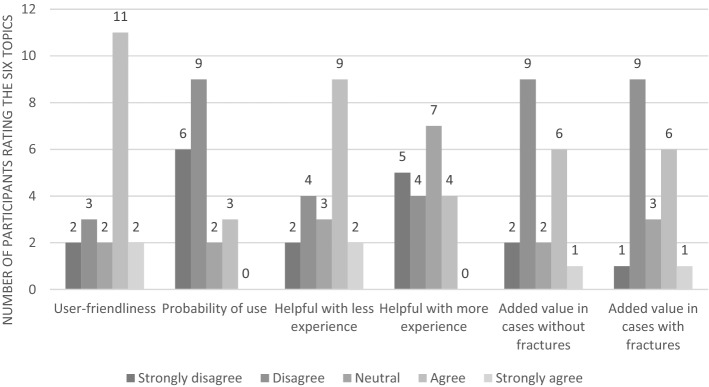
Overview of rated opinion of the RCWC.

The user-friendliness of both classifications was rated using six theses and is depicted in Figs. 1 and 2. The GAWC was rated less user-friendly than the RCWC. Participants considered the RCWC’s categories for each wound assessment parameter easier to choose from than those of the GAWC. This was reflected by faster evaluation per wound with the RCWC and the struggle of some participants to assign a GAWC type when not all wound characteristics reflected one particular GAWC type. Some participants assigned the most severe GAWC type they had chosen for one of the wound assessment parameters, while others assigned the average GAWC type. Participants stated they could bring more nuance to wound assessment and description without using a classification. They also explained that the GAWC better reflected their current (non-systematic) wound assessment method than the RCWC.

Participants considered the RCWC more applicable to severe wounds or gunshot wounds and limited in its application, as these wounds occur less frequently at their hospital. Furthermore, they stated that the RCWC does not effectively discriminate injury to a vital structure well in all acute wounds. For example, many participants were conflicted, as within the RCWC, only damage to the spinal cord or brain is deemed injury to a vital neurological structure, thereby excluding peripheral nerve injury. Participants argued that peripheral nerve injury could have major consequences for the patient and should therefore be considered damage to a vital structure.

Both classifications were regarded as more helpful when being less experienced as a healthcare professional. The GAWC’s added value was considered greater in acute wounds with fractures than without. The RCWC’s added value in acute wounds appeared independent of the presence of fractures.

## Discussion

This multicentre cross-sectional survey study is the first to evaluate the currently used classifications in acute wound assessment and analyse the interobserver variability of the GAWC and RCWC in acute wounds in the ED. Fifty percent of the participants never use a classification for assessing acute wounds at Dutch EDs. Currently, no standard protocol exists for acute wound assessment, and it is common practice to describe the wound, presence of a fracture, and neurovascular injury in an electronic patient file occasionally including a picture. The interobserver variability of the GAWC (κ = 0.46) and RCWC grades (κ = 0.56) is considered moderate, and good for the RCWC types (κ = 0.69), suggesting the RCWC is at least as suitable for acute wound assessment as the GAWC. Only minor differences were observed between emergency physicians and emergency medicine residents, suggesting both classifications are equally reliable when used by these groups of healthcare professionals. Both classifications were considered helpful for acute wound assessment when less experienced. However, their user-friendliness was considered moderate.

The Dutch guideline on wound care recommends using a standard tool for acute wound assessment and advocates using the RYB system with the TIME model for a uniform and systematic wound care process^4^. Our results show no wound classification is routinely used in the Netherlands. The influence of this guideline on daily practice is thus minimal.

The moderate agreement of the GAWC for acute wounds (κ = 0.46) is consistent with previous studies performed on patients with open fractures (κ = 0.53 and κ = 0.44)^13,14^, indicating the GAWC is as reliable in acute wounds without fractures. A small difference in kappa values, in slight disadvantage of our study (κ = 0.46), was found in open fractures in the study by Horn et al. (κ = 0.53)^13^. This may be because the GAWC is usually applied during surgery when tissue damage is often more visible and easier to assess^24,25^, whereas our study setting for first wound assessment concerned the ED, resembling current practice within the Netherlands. Comparisons of the level of agreement between physicians and residents show varying results. Horn et al. found similar agreement between orthopaedic surgeons and residents, as the differences in kappa values (κ = 0.63 and κ = 0.49, respectively) were not statistically significantly different^13^. Our study (κ = 0.45; κ = 0.47) and studies of other classifications show a similar or higher agreement among residents compared to attendings, although these were not statistically analysed^11,12^. The aforementioned subtle differences in agreement may be due to individual differences in the content and phase of medical training.

This study has several strengths and limitations. One of its strengths encompasses the variety of the study population, which comprises emergency physicians with varying experience levels from multiple hospitals. It accurately reflects the healthcare professionals who perform a first assessment of acute wounds and may use a wound classification. Wound variety of the fictitious cases was representative of that seen in Dutch EDs, considering severity and cause, endorsing the study outcome generalisability within the Netherlands. Generalisability for other countries may be more difficult due to differences in education and national hospital protocols. Another limitation is that wound assessment is probably more difficult and less accurate when using fictitious cases and pictures (due to COVID-19 restrictions), possibly resulting in a lower level of agreement. Moreover, good interobserver variability may not reflect assignment of the correct type or grade, as kappa analyses only show interobserver agreement. Furthermore, this study validated the GAWC and RCWC for acute civilian soft tissue injuries, while the GAWC was originally designed for open fractures and the RCWC for war wounds.

The authors recommend using the GAWC in the assessment of acute wounds with an underlying fracture for a uniform approach. The RCWC has been validated in conflict settings and might also be useful for acute wounds in a civilian setting. It can be of additional value in major injuries with extensive tissue damage. Ideally, each wound category would be related to a management approach to benefit most from using a classification system. The GAWC is already used to determine the appropriate antibiotic treatment for open fractures^7,26–28^. However, no validated treatment algorithm exists for acute wounds without underlying fractures. Additionally, the RCWC is used to estimate injury severity and subsequently guide resources and estimate the complexity of repairing the damaged structures^19,20^, which is not yet validated in a civilian setting.

## Conclusions

In conclusion, no standard wound classification is currently in practice for acute wound assessment in Dutch emergency departments. Healthcare professionals are reserved to use existing classifications due to their moderate user-friendliness and because most healthcare professionals hold on to their own systematic approach. However, the interobserver variability of the GAWC and RCWC in acute wounds is promising. These classifications facilitate uniform communication and could help less experienced healthcare providers perform a systematic wound assessment. It is recommended to raise awareness of existing wound classifications and encourage their routine application in the ED. Future prospective research is recommended to assess whether treatment strategies can be linked to categories of the GAWC and RCWC in acute wounds, if such treatment algorithm results in improved patient outcomes, and if the use of such treatment algorithm results in more cost-effective wound care.

## Supplementary Information

Supplementary Information 1.

Supplementary Information 2.

Supplementary Information 3.

## Data Availability

The datasets used or analysed during the current study are available from the corresponding author on reasonable request.

## Abbreviations

AO: Arbeitsgemeinschaft Osteosynthesefragen
CI: Confidence interval
ED: Emergency department
GAWC: Gustilo Anderson Wound Classification
IQR: Interquartile range
OTA: Orthopaedic Trauma Association
RCWC: Red Cross Wound Classification
RYB: Red Yellow Black

## References

[1] Letsel Informatie Systeem. SEH Behandelingen lichamelijk letsel 2016: VeiligheidNL; 2017. https://www.volksgezondheidenzorg.info/onderwerp/acute-zorg/cijfers-context/gebruik-acute-zorg#node-gebruik-seh-naar-diagnose. Accessed 16 April 2020.

[2] Kumar S, Leaper DJ (2005). Classification and management of acute wounds. Surgery..

[3] LeBlanc K, Baranoski S, Holloway S, Langemo D, Regan M (2014). A descriptive cross-sectional international study to explore current practices in the assessment, prevention and treatment of skin tears. Int. Wound J..

[4] Nederlandse Vereniging voor Heelkunde. Richtlijn Wondzorg. Utrecht 2013. 226 p.

[5] Cuzzell JZ (1988). The new RYB color code. Am. J. Nurs..

[6] Ubbink DT, Brolmann FE, Go PM, Vermeulen H (2015). Evidence-based care of acute wounds: A perspective. Adv. Wound Care (New Rochelle)..

[7] Gustilo RB, Anderson JT (1976). Prevention of infection in the treatment of one thousand and twenty-five open fractures of long bones: Retrospective and prospective analyses. J. Bone Joint Surg. Am..

[8] Rüedi TP, Buckley RE, Moran CG (2007). AO Principles of Fracture Management.

[9] Orthopaedic Trauma Association: Open Fracture Study G (2010). A new classification scheme for open fractures. J. Orthop. Trauma..

[10] Tscherne H, Oestern HJ (1982). A new classification of soft-tissue damage in open and closed fractures (author's transl). Unfallheilkunde.

[11] Valderrama-Molina CO, Estrada-Castrillon M, Hincapie JA, Lugo-Agudelo LH (2014). Intra- and interobserver agreement on the Oestern and Tscherne classification of soft tissue injury in periarticular lower-limb closed fractures. Colomb. Med. (Cali.)..

[12] Agel J, Evans AR, Marsh JL, Decoster TA, Lundy DW, Kellam JF (2013). The OTA open fracture classification: A study of reliability and agreement. J. Orthop. Trauma..

[13] Horn BD, Rettig ME (1993). Interobserver reliability in the Gustilo and Anderson classification of open fractures. J. Orthop. Trauma..

[14] Ghoshal A, Enninghorst N, Sisak K, Balogh ZJ (2018). An interobserver reliability comparison between the Orthopaedic Trauma Association's open fracture classification and the Gustilo and Anderson classification. Bone Joint J..

[15] Coupland, R.M. The Red Cross Wound Classification: International Committee of the Red Cross. (1991). 18.

[16] Percival NJ (2002). Classification of wounds and their management. J. Surg..

[17] Greenhalgh DG (2019). Management of burns. N. Engl. J. Med..

[18] Cernak I, Savic J, Zunic G, Pejnovic N, Jovanikic O, Stepic V (1999). Recognizing, scoring, and predicting blast injuries. World J. Surg..

[19] Coupland RM (1992). The Red Cross classification of war wounds: The E.X.C.F.V.M. scoring system. World J. Surg..

[20] Giannou, C., Baldan, M. War Surgery Working with Limited Resources in Armed Conflict and Other Situations of Violence Geneva: International Committee of the Red Cross (2010).

[21] Vassallo D, McAdam G (1995). Modification to Red Cross wound classification. Injury.

[22] Fleiss JL (1971). Measuring nominal scale agreement among many raters. Psychol. Bull..

[23] Landis JR, Koch GG (1977). The measurement of observer agreement for categorical data. Biometrics.

[24] Melvin JS, Dombroski DG, Torbert JT, Kovach SJ, Esterhai JL, Mehta S (2010). Open tibial shaft fractures: I. Evaluation and initial wound management. J. Am. Acad. Orthop. Surg..

[25] Okike K, Bhattacharyya T (2006). Trends in the management of open fractures. A critical analysis. J. Bone Joint Surg. Am..

[26] Gustilo RB, Mendoza RM, Williams DN (1984). Problems in the management of type III (severe) open fractures: A new classification of type III open fractures. J. Trauma..

[27] Nederlandse Vereniging voor Heelkunde. Richtlijn Open onderbeenfractuur. Utrecht (2016). 136.

[28] Sharareh, B. Open Fractures Management: Orthobullets [updated May 3, 2020]. https://www.orthobullets.com/trauma/1004/open-fractures-management. Accessed 27 July 2020.

