# Supplementary material for: A multicentre cross-sectional survey study on acute wound classification in the emergency department and its interobserver variability
Source: Sci Rep. 2022 Jun 14;12:9901. doi: 10.1038/s41598-022-13221-1 (PMC9196857; doi:10.1038/s41598-022-13221-1)
Supplement: Supplementary file 1 — Supplementary Information 1. [file 41598_2022_13221_MOESM1_ESM.pdf]

Appendix 1 – Gustilo Anderson wound classification

Open fractures were originally divided into three categories as listed below.[1]

Type I:	Wound <1 cm; clean
Type II:	Wound >1 cm without extensive soft tissue damage, flaps, or avulsions
Type III:	Open segmental fracture, open fracture with extensive soft tissue damage, or a traumatic amputation

Type III comprised special categories, comprising gunshot injuries, farm injuries resulting in an open fracture, and open fractures accompanied by a vascular injury requiring repair.[1]

In 1984, Gustilo et al. implemented a subdivision into the former type III category, as much diversity was found regarding the severity and prognosis of these open fractures.[2] Currently, the GAWC is still applied, using the subdivision listed below.[2]

Type IIIA:	Adequate soft tissue coverage despite extensive soft tissue laceration or flaps, or high-energy trauma irrespective of the wound size
Type IIIB:	Extensive soft tissue injury loss with periosteal stripping and bone exposure, usually associated with massive contamination
Type IIIC:	Open fracture associated with arterial injury requiring repair

Table 1 shows the complete GAWC with the variables requiring assessment.

Table 1: Gustilo Anderson wound classification[3]

	Type I	Type II	Type IIIA	Type IIIB	Type IIIC
**Energy**	Low	Moderate	High	High	High
**Wound size**	≤1 cm	1-10 cm	Usually >10 cm	Usually >10 cm	Usually >10 cm
**Soft tissue damage**	Minimal	Moderate	Extensive	Extensive	Extensive
**Contamination**	Clean	Moderate	Extensive	Extensive	Extensive
**Fracture pattern**	Simple fracture pattern with minimal comminution	Moderate comminution	Severe comminution or segmental fractures	Severe comminution or segmental fractures	Severe comminution or segmental fractures
**Periosteal stripping**	No	No	Yes	Yes	Yes
**Skin coverage**	Local	Local	Local	Requires free tissue flap or rotational flap coverage	Typically requires flap coverage
**Neurovascular injury**	Normal	Normal	Normal	Normal	Exposed fracture with arterial damage requiring repair

References

1. Gustilo RB, Anderson JT. Prevention of infection in the treatment of one thousand and twenty-five open fractures of long bones: retrospective and prospective analyses. J Bone Joint Surg Am. 1976;58(4):453-8.

2. Gustilo RB, Mendoza RM, Williams DN. Problems in the management of type III (severe) open fractures: a new classification of type III open fractures. J Trauma. 1984;24(8):742-6.

3. Torchia M, Taylor BC. Gustilo Classification: Orthobullets; [updated June 25, 2018May 4, 2020]. Available from: https://www.orthobullets.com/trauma/1003/gustilo-classification.
