# Supplementary material for: A multicentre cross-sectional survey study on acute wound classification in the emergency department and its interobserver variability
Source: Sci Rep. 2022 Jun 14;12:9901. doi: 10.1038/s41598-022-13221-1 (PMC9196857; doi:10.1038/s41598-022-13221-1)
Supplement: Supplementary file 2 — Supplementary Information 2. [file 41598_2022_13221_MOESM2_ESM.pdf]

__Additional file 2 – Red Cross wound classification__

In the RCWC, some anatomical features are used to score a penetrating wound to rapidly assess its significance[1]. This rapid assessment facilitates triage in the field or ED and communication between the outpatient department and operating theatre. The size, cavity, and fracture (if any) of the wound are used to it. The injured structures are used to type the wound. The type of weapon or projectile that caused the wound is not a factor. Variables used are as follows:[1]

	**Assessment**
**E (entry wound)**	The maximal diameter of the entry wound in cm
**X (exit wound)**	The maximal diameter of the exit wound in cm (0 cm if no exit wound is present)
**C (cavity)**	Can the cavity of the wound take two fingers before surgical excision?<br>**C = 0**: No<br>**C = 1**: Yes
**F (fracture)**	Are any bones fractured?<br>**F = 0**: No fracture<br>**F = 1**: Simple fracture, hole, or insignificant comminution<br>**F = 2**: Clinically significant comminution
**V (vital structure)**	Are dura, pleura, peritoneum or major peripheral vessels injured?<br>**V = 0**: No vital structure injured<br>**V = N** (neurological): Penetration of the dura of the brain or spinal cord<br>**V = T** (thorax/trachea): Penetration of the pleura or of the larynx/trachea in the neck<br>**V = A** (abdomen): Penetration of the peritoneum<br>**V = H** (haemorrhage): Injury to a major peripheral blood vessel down to brachial or popliteal arteries, or carotid artery in the neck
**M (metallic body)**	Are bullets or fragments visible on radiographs?<br>**M = 0**: No<br>**M = 1**: Yes, one metallic body<br>**M = 2**: Yes, multiple metallic bodies

Wounds are graded according to their severity, which is related to the degree of kinetic energy transfer of the projectile to the body tissue.[1-4]

Grade 1: $E + X < 10$ cm with C0 and F0 or F1

Grade 2: $E + X < 10$ cm with C1 or F2

Grade 3: $E + X \geq 10$ cm with C1 or F2

Subsequently, wounds are categorised according to the injured tissue structures.[1-4]

Type ST: Soft tissue wounds: F0 and V0

Type F: Wounds with fractures: F1 or F2, and V0

Type V: Vital wounds putting the patient's life at risk: F0 and V = N, T, A, or H

Type VF: Wounds with fractures and involving vital structures putting life or limb at risk: F1 or F2 and V = N, T, A, or H

References

1. Giannou C, Baldan M. War Surgery Working With Limited Resources in Armed Conflict and Other Situations of Violence Geneva: International Committee of the Red Cross; 2010.

2. Cernak I, Savic J, Zunic G, Pejnovic N, Jovanikic O, Stepic V. Recognizing, scoring, and predicting blast injuries. World J Surg. 1999;23(1):44-53.

3. Coupland RM. The Red Cross classification of war wounds: the E.X.C.F.V.M. scoring system. World J Surg. 1992;16(5):910-7.

4. Vassallo D, McAdam G. Modification to Red Cross wound classification. Injury. 1995;26(2):131-2.
