# Supplementary material for: A multicentre cross-sectional survey study on acute wound classification in the emergency department and its interobserver variability
Source: Sci Rep. 2022 Jun 14;12:9901. doi: 10.1038/s41598-022-13221-1 (PMC9196857; doi:10.1038/s41598-022-13221-1)
Supplement: Supplementary file 3 — Supplementary Information 3. [file 41598_2022_13221_MOESM3_ESM.pdf]

Additional file 3 – Questionnaire wound classifications

This questionnaire serves as visual support during the online oral questionnaire. Preceding the appointment for the online oral questionnaire, you do not have to fill in this questionnaire.

First some general questions and questions regarding the currently used classifications.

1. **In which hospital are you employed?**
    1) Jeroen Bosch hospital, Den Bosch
    2) Elisabeth TweeSteden hospital, Tilburg
    3) Rijnstate, Arnhem
    4) Hospital Rivierenland, Tiel
    5) Slingeland hospital, Doetinchem
    6) Streekziekenhuis Koningin Beatrix, Winterswijk
    7) Hospital Gelderse Vallei, Ede
    8) Canisius Wilhelmina hospital, Nijmegen
    9) Radboudumc, Nijmegen

2. **What is your function?**
    1) Emergency physician
    2) Emergency medicine resident

3. **How many years of experience do you have in total as an emergency medicine resident and emergency physician combined?**

Number of years:

4. **Which classification do you currently use most for acute wounds at the emergency department?**
    0) None
    1) Gustilo Anderson classification
    2) Red Cross wound classification
    3) Red Yellow Black system
    4) Degree of contamination
    5) TIME model
    6) Other:

5. **What is the reason for the use of this classification system?**
    1) Not applicable
    2) Support of wound assessment
    3) For a systematic assessment
    4) Support of the estimation of the prognosis and/or treatment
    5) Other:

6. **Were you already familiar with the Gustilo Anderson classification before reading the informational document?**
    1) Yes
    2) No

7. **How often do you use the Gustilo Anderson classification?**
    1) Never
    2) <1 time per 6 months
    3) <1 time per month

    4) <1 time per week
    5) 1 time or more times per week
    6) Daily
    7) Not applicable

8. **Were you already familiar with the Red Cross wound classification before reading the informational document?**
    1) Yes
    2) No

9. **How often do you use the Red Cross wound classification?**
    1) Never
    2) <1 time per 6 months
    3) <1 time per month
    4) <1 time per week
    5) 1 time or more times per week
    6) Daily
    7) Not applicable

Next, 10 patient cases with acute wounds (with and without fractures) are presented. You are going to assess these wounds using both the Gustilo Anderson and the Red Cross wound classification. Step by step you will assess the characteristics of each wound in order to type the wound with the Gustilo Anderson classification and grade and type the wound with the Red Cross wound classification.

All wounds are first assessed using the Gustilo Anderson classification and are then assessed using the Red Cross wound classification. The informational document you received regarding the classifications can be used to aid the assessment of the patient cases. If there is no fracture, you assign the type of the Gustilo Anderson classification that fits best, regardless of the parameter 'fracture pattern'.

Note: The following questions are answered for all ten patient cases.

<u>**Gustilo Anderson classification**</u>

1. **What is the estimated degree of energy transfer to the damaged tissue?**
    1) Low
    2) Moderate
    3) High

2. **What is the maximal diameter of the wound (in cm, accurate to 1 decimal point)?**
    1) Maximal diameter:

3. **What is the estimated soft tissue damage?**
    1) Minimal
    2) Moderate
    3) Extensive

4. **What is the estimated contamination of the wound?**
    1) Clean
    2) Moderate contamination
    3) Extensive contamination

5. **Is there a fracture and how would you assess the type of fracture?**

1) None
2) Simple fracture with minimal comminution
3) Moderate comminution
4) Severe comminution or segmental fracture

6. **Is there periosteal stripping?**
    1) Yes
    2) No

7. **What is the preferred method for skin coverage?**
    1) Local
    2) Requires either free tissue flap or rotational flap coverage
    3) Requires free tissue flap coverage

8. **Is neurovascular injury present?**
    1) No
    2) Exposed fracture with arterial damage requiring repair

9. **Which type of the Gustilo Anderson classification would you assign to the wound according to the answers above?**
    1) Type I
    2) Type II
    3) Type III A
    4) Type III B
    5) Type III C

<u>Red Cross wound classification</u>

1. **What is the maximal diameter of the entry wound (in cm, accurate to 1 decimal point)?**
    0) Maximal diameter:

2. **What is the maximal diameter of the exit wound (in cm, accurate to 1 decimal point)?**
    0) Maximal diameter:

3. **Do you estimate that the cavity of the wound is as wide as or wider than 2 fingers before surgical excision?**
    0) No
    1) Yes

4. **Are any bones fractured and how would you asses this fracture?**
    0) No fracture
    1) Simple fracture, hole or insignificant comminution
    2) Clinically significant comminution

5. **Are any vital structures damaged, if so, which?**
    0) No vital structure injured
    1) Neurological: penetration of the dura of the brain or spinal cord
    2) Thorax/trachea: penetration of the pleura or of the larynx/trachea in the neck
    3) Abdomen: penetration of the peritoneum
    4) Haemorrhage: injury to a major peripheral blood vessel down to brachial or popliteal arteries, or carotid artery in the neck

6. **Are bullets or fragments visible on radiographs?**

0) No
1) Yes, one metallic body
2) Yes, multiple metallic bodies

7. **Which degree of the Red Cross wound classification would you assign to the wound according to the answers above?**
   1) Grade 1
   2) Grade 2
   3) Grade 3

8. **Which type of the Red Cross wound classification would you assign to the wound according to the answers above?**
   1) Type ST
   2) Type F
   3) Type V
   4) Type VF

Lastly, a few theses are proposed regarding the user-friendliness of the Gustilo Anderson classification and the Red Cross wound classification. You will be scoring them on a scale from 1 to 5, with 1 ‘strongly disagree’ and 5 ‘strongly agree’.

<u>Gustilo Anderson classification</u>

1. **The Gustilo Anderson classification is user-friendly.**
   1) Strongly disagree
   2) Disagree
   3) Neutral
   4) Agree
   5) Strongly agree

2. **It is likely that I will use the Gustilo Anderson classification for the assessment of acute wounds.**
   1) Strongly disagree
   2) Disagree
   3) Neutral
   4) Agree
   5) Strongly agree

3. **The Gustilo Anderson classification is helpful for acute wounds when the healthcare professional is less experienced, for example as an emergency medicine resident at the ED.**
   1) Strongly disagree
   2) Disagree
   3) Neutral
   4) Agree
   5) Strongly agree

4. **The Gustilo Anderson classification is helpful for acute wounds when the healthcare professional is more experienced, for example as an emergency physician at the ED.**
   1) Strongly disagree
   2) Disagree
   3) Neutral
   4) Agree
   5) Strongly agree

5. **The Gustilo Anderson classification is of added value in the assessment of acute wounds without fractures.**
   1) Strongly disagree
   2) Disagree
   3) Neutral
   4) Agree
   5) Strongly agree

6. **The Gustilo Anderson classification is of added value in the assessment of acute wounds with fractures.**
   1) Strongly disagree
   2) Disagree
   3) Neutral
   4) Agree
   5) Strongly agree

<u>Red Cross wound classification</u>

1. **The Red Cross wound classification is user-friendly.**
   1) Strongly disagree
   2) Disagree
   3) Neutral
   4) Agree
   5) Strongly agree

2. **It is likely that I will use the Red Cross wound classification for the assessment of acute wounds.**
   1) Strongly disagree
   2) Disagree
   3) Neutral
   4) Agree
   5) Strongly agree

3. **The Red Cross wound classification is helpful for acute wounds when the healthcare professional is less experienced, for example as an emergency medicine resident at the ED.**
   1) Strongly disagree
   2) Disagree
   3) Neutral
   4) Agree
   5) Strongly agree

4. **The Red Cross wound classification is helpful for acute wounds when the healthcare professional is more experienced, for example as an emergency physician at the ED.**
   1) Strongly disagree
   2) Disagree
   3) Neutral
   4) Agree
   5) Strongly agree

5. **The Red Cross wound classification is of added value in the assessment of acute wounds without fractures.**
   1) Strongly disagree

2) Disagree
3) Neutral
4) Agree
5) Strongly agree

6. **The Red Cross wound classification is of added value in the assessment of acute wounds with fractures.**
    1) Strongly disagree
    2) Disagree
    3) Neutral
    4) Agree
    5) Strongly agree
